# Variation in Sodic Soil Bacterial Communities Associated with Different Alkali Vegetation Types

**DOI:** 10.3390/microorganisms9081673

**Published:** 2021-08-06

**Authors:** Andrea K. Borsodi, Márton Mucsi, Gergely Krett, Attila Szabó, Tamás Felföldi, Tibor Szili-Kovács

**Affiliations:** 1 Department of Microbiology, ELTE Eötvös Loránd University, Pázmány P. Sétány 1/C, H-1117 Budapest, Hungary; mucsi.marton@atk.hu (M.M.); gergely.krett@ttk.elte.hu (G.K.); tamas.felfoldi@ttk.elte.hu (T.F.); 2 Institute of Aquatic Ecology, Centre for Ecological Research, Karolina út 29, H-1113 Budapest, Hungary; szabo.attila@ecolres.hu; 3 Institute for Soil Sciences, Centre for Agricultural Research, Herman Ottó út 15, H-1022 Budapest, Hungary

**Keywords:** 16S rRNA gene, bacterial diversity, catabolic activity, cultivation, Pannonian steppe, pyrosequencing

## Abstract

In this study, we examined the effect of salinity and alkalinity on the metabolic potential and taxonomic composition of microbiota inhabiting the sodic soils in different plant communities. The soil samples were collected in the Pannonian steppe (Hungary, Central Europe) under extreme dry and wet weather conditions. The metabolic profiles of microorganisms were analyzed using the MicroResp method, the bacterial diversity was assessed by cultivation and next-generation amplicon sequencing based on the 16S rRNA gene. Catabolic profiles of microbial communities varied primarily according to the alkali vegetation types. Most members of the strain collection were identified as plant associated and halophilic/alkaliphilic species of *Micrococcus*, *Nesterenkonia*, *Nocardiopsis*, *Streptomyces* (Actinobacteria) and *Bacillus*, *Paenibacillus* (Firmicutes) genera. Based on the pyrosequencing data, the relative abundance of the phyla Proteobacteria, Actinobacteria, Acidobacteria, Gemmatimonadetes and Bacteroidetes also changed mainly with the sample types, indicating distinctions within the compositions of bacterial communities according to the sodic soil alkalinity-salinity gradient. The effect of weather extremes was the most pronounced in the relative abundance of the phyla Actinobacteria and Acidobacteria. The type of alkali vegetation caused greater shifts in both the diversity and activity of sodic soil microbial communities than the extreme aridity and moisture.

## 1. Introduction

Approximately 7% of the terrestrial habitats are affected by salt around the world [1], of which Solonchaks (high soluble salt soils) and Solonetz (with a high content of exchangeable Na^+^) soils are estimated to cover about 260 and 135 million ha by the IUSS Working Group [2], respectively. Unlike coastal areas where sodium chloride accumulation influences salt-affected soils, the dominance of sodium sulphate and sodium carbonate affects the soils in continental areas [3].

Soil salinity is the main limiting and selective factor for the living world by creating an alkaline-saline soil environment in agricultural areas and natural habitats as well. Enhanced salt content and pH affect plant growth, reducing photosynthesis and transpiration, with a directly effect on plant nutrient availability [4]. Plants adapted to these environments have developed multiple biochemical pathways to tolerate ionic and drought stress [5], although living alone may means plants are not able to survive in extreme conditions, while rhizosphere microorganisms including archaea, bacteria (plant growth promoting rhizobacteria) and fungi (especially arbuscular mycorrhiza fungi) can alleviate the environmental stress and promote plant growth by increasing the availability of water, nitrogen and minerals from the soil [6]. Thus, the rhizosphere microbiota has an essential role in the shape and activity of vegetation in sodic soils, which has been sparsely studied [4]. High salinity and sodicity coupled with increased pH have selective effects on microbial community composition and activity [7]. Studying the diversity of bacterial and archaeal communities along a natural salinity gradient, an opposite trend was observed in the hypersaline soils [8]. The highest salinity sites were dominated by archaeal populations, whereas bacterial diversity decreased along the increasing salinity gradient. Soil-groundwater salinity was found as one of the most important factors that determines the composition and richness of soil microbial communities in a central Italian coastal system [9]. The meta-analysis of the publicly available 16S rRNA gene sequences identified from saline and hypersaline soils revealed that Proteobacteria, Actinobacteria, Firmicutes, Acidobacteria, Bacteroidetes and Chloroflexi predominated the bacterial communities [10]. Besides the phylum Euryarchaeota, Proteobacteria was also the most abundant in the saline soils of Qarhan Salt Lake (China), followed by the phyla Bacteroidetes and Gemmatimonadetes, as revealed by high-throughput sequencing [11]. 

A part of the Pannonian steppe, located in the Carpathian Basin, Europe has saline-sodic soils too. Salinization generally appears in patches in the whole area; therefore, only a few centimeters of soil unevenness can result in significant difference in surface salt concentrations [12]. 

The alkali vegetation type is a mostly loose in structure, open herbaceous plant communities with only a few species [12,13]. The salt-affected Pannonian steppe represents outstanding geological, hydrological, botanical and zoological value; therefore, it is under nature protection (Management of Natura 2000 habitats; Directive 92/43/EEC on the conservation of natural habitats and wild fauna and flora).

The physical-chemical background and hydrogeological processes of salt accumulation and sodicity as well as their effects on the vegetation types of the Pannonian steppe have been studied and recorded [12,13,14,15,16,17], but their relationship with the activity and diversity of microorganisms are far less examined and known. This fact has drawn our attention to exploring and comparing the microbiota of these unique and vulnerable habitats to explore bacterial communities and cultivable bacteria that may alleviate salinity and drought stress of plants.

In the present study, our aim was to reveal (i) the effects of four alkali vegetation types along with the soil properties (salinity and alkalinity gradient) on the microbial catabolic activity profiles and bacterial diversity of the rhizosphere and (ii) if they differ under extreme dry and wet soil conditions.

## 2. Materials and Methods

### 2.1. Description of Sampling Sites

**Figure 1:**
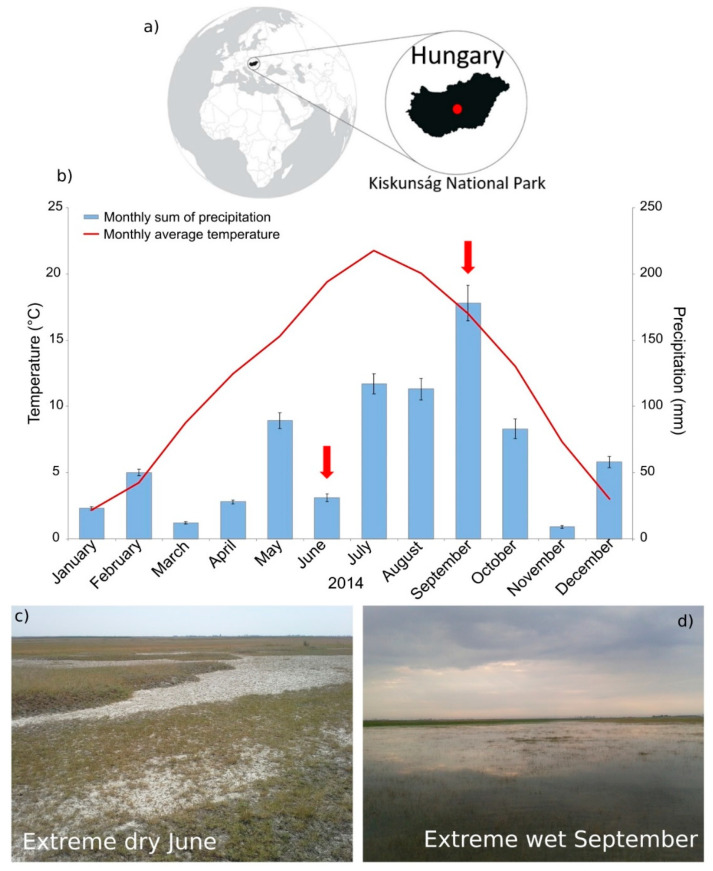
(**a**) Location of sampling sites in Europe and in the area of Kiskunság National Park, Hungary. The maps in the figure were redrawn after Anda et al. 2020 [18]. (**b**) Changes in weather conditions in the year of sampling based on monthly average air temperature (°C) and monthly sum of precipitation (mm) in the Pannonian steppe, Kiskunság NP, Hungary (Red arrows mark sampling dates.). Error bars show the standard deviations calculated from the daily differences of precipitations. Data obtained from the public database of the Hungarian Meteorological Services. Photos show the *Puccinellia* sward sampling site AP during (**c**) the extreme dry June and (**d**) the extreme wet September.

Sampling sites can be found in the Kiskunság National Park (KNP), Hungary located in the Danube-Tisza Interfluve (Figure 1a) at approximately 100 m above sea level. Solonetz is the predominant soil type, but Solonchak salt-affected soils also occur in mosaic patterns [12]. The studied soils are covered by four distinct types of alkali steppe vegetation: (1) bare spot (AL), (2) *Puccinellia* sward (AP), (3) *Artemisia* alkali steppe (AA), (4) *Achillea* alkali steppe (AF), detailed in Table 1.

Most of the salt steppes in the region represent semi-natural habitats where biological diversity is maintained in conjunction with human activities. The natural grassy vegetation covered soils are not cultivated, and their maintenance is provided mainly by grazing. A more detailed description of the Hungarian alkali vegetation is available in the papers of Molnár and Borhidi [12].

Based on particle size distribution, the soil texture is loam at the AL and AP sampling sites, clay loam at the AA site and silty loam at the AF site.

### 2.2. Weather Conditions, Sampling and Sample Processing

In the Pannonian steppe, the climate is temperate, with 10 °C annual mean air temperature (min. −2 °C in January and max. +21 °C in July), the average annual sum of precipitation is 527 mm, and the mean annual potential evaporation is 900 mm. The average depth of groundwater level is 1.6 m, with a range of 0.6–2.3 m [19]. 

Following a drier than usual spring, the monthly sum of precipitation (31 mm) in June 2014 was only 54% of the long-term annual average (Figure 1b), and the air temperature showed an anomaly of about +5 °C compared to the average for previous years. The bare spot and *Puccinellia* sward sampling sites were completely dried out, the surface of the soils cracked in the extremely dry and warm period (Figure 1c). The monthly sum of precipitation (178 mm) in September 2014 was, however, more than two times higher than the long-term annual average (Figure 1b), and the 16.8 °C monthly average air temperature was also above the long-term average. In that extremely wet period, the bare spot and *Puccinellia* sward sampling sites were waterlogged or even flooded in the extremely wet period (Figure 1d).

Sodic soil samples were taken at the same sampling sites under both the extremely dry (June 2014) and wet (September 2014) weather conditions. In each sampling site, three representative plots were appointed, where three further individual cores were randomly collected from the surface (0–15 cm) soil horizons. Approximately 500 g of each soil sample was pooled into sterile jars and transported in a cooling box (6–8 °C) to the laboratory. Rhizosphere soil samples for the cultivation of bacteria and metagenome analyses were taken from the root adhering soils using a sterile spatula and placed in sterile tubes, then transported in a cooling box to the lab. Prior to the laboratory examinations, samples were homogenized, and the visible roots and other plant residues were removed. Altogether, 24 soil samples were obtained and divided into subsamples for soil moisture, chemical and microbiological analyses.

### 2.3. Physical and Chemical Characterization of the Soil Samples

The gravimetric moisture content of the soil samples was measured after drying at 105 °C for 24 h. Soil chemical analyses were performed on air-dried and sieved (<2 mm) soil samples according to the Hungarian soil standard methodology, such as soil organic carbon content by dichromate oxidation; lime (CaCO_3_) content with a calcimeter; soil pH with a pH-meter and glass electrodes in a 1:2.5 soil to water suspension, and electrical conductivity in the water saturated soil paste (EC) by an EC-meter [20].

### 2.4. Catabolic Activity Profiles

MicroResp technique was used to evaluate the catabolic activity of the soil microbial communities [21]. The water content of the soil samples was set to 50% of their water holding capacities, then a 5-day preincubation was applied. For the MicroResp measurements, 15 different substrates and ultrapure water were distributed in 6 repetitions. The following substrates were used: D-galactose (Gal), trehalose (Tre), L-arabinose (Ara), D-glucose (Glc) and D-fructose (Fru) in 80 mg mL^−1^, citric acid (Cit), DL-malic acid (Mal), Na-succinate (Suc), L-alanine (Ala) and L-lysine (Lys) in 40 mg mL^−1^, L-glutamine (Gln) in 20 mg mL^−1^, and L-leucine (Leu), L-arginine (Arg), protocatechuic acid (Proc) and L-glutamic acid (Glu) in 12 mg mL^−1^. The pH of the substrate solution was adjusted between 5.5 and 6.0 by dropping in 1 mol L^−1^ HCl or NaOH solutions. The deep-well plates were left open for one hour after addition of substrate solutions to avoid interference with any possible abiotic CO_2_ production. The plates were incubated for 5 h at 25 °C, the detector plates were read with a plate reader (Anthos 2010, Biochrom Ltd., Cambridge, UK) at 570 nm immediately before and after the incubation. The respiration rates (µg CO_2_-C g soil^−1^ h^−1^) were calculated from the absorbance values as described by the MicroResp^TM^ Technical Manual.

### 2.5. Cultivation-Based Bacteriological Examinations

The cultivable bacterial diversity was explored using Horikoshi alkaline (DSMZ 940) and modified R2A (DSMZ 830) media (www.dsmz.de (accessed on 3 August 2021)) as described by Borsodi et al. [22].

The methodology of DNA extraction, ARDRA (Amplified Ribosomal DNA Restriction Analysis) and 16S rRNA gene-based identification of bacterial strains was the same as previously [22].

DNA derived from the representative bacterial strains was sequenced with 27f primer using the Sanger method by the LGC Genomics (Berlin, Germany).

The obtained sequences were compared to 16S rRNA gene sequences available in the EzBioCloud database [23]. Phylogenetic and molecular evolutionary analyses were conducted using MEGA version X [24]. The evolutionary history was inferred using the maximum likelihood method based on the general time reversible model [25]. 

The 16S rRNA gene sequences were submitted to the GenBank under the accession numbers LR216720-LR216777 for bacterial strains.

### 2.6. Community DNA Extraction and Pyrosequencing

Community DNA was isolated from the soil samples using the Power Soil DNA isolation kit (MoBio, Carlsbad, CA, USA) according to the manufacturer’s instructions.

The whole procedure was the same as previously described in detail [26]. Briefly, the V3–V4 region of the 16S rRNA gene was amplified using Bacteria domain-specific primers and the PCR products of the three parallel reactions originated from the same sampling sites were pooled and sequenced as a composite sample on a Roche GS Junior platform. The bioinformatic analysis of the resulting sequence reads were carried out with mothur [27]. Raw sequence reads are available in the NCBI Sequence Read Archive as BioProject accession PRJNA316799.

### 2.7. Statistical Analyses

**Figure 2:**
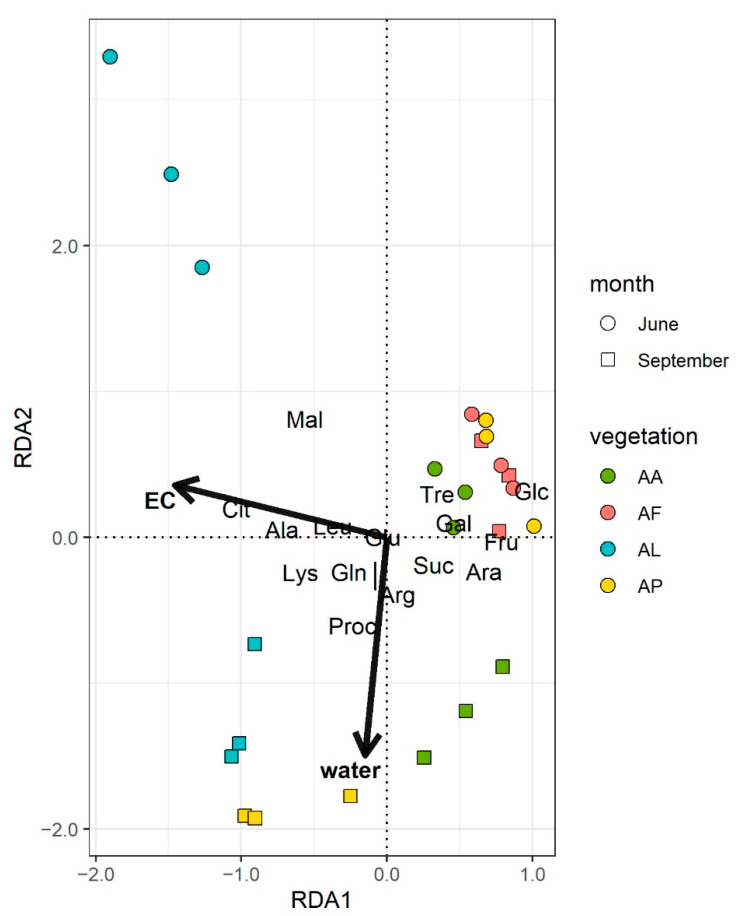
Redundancy analysis plot of the final model for the catabolic profiles. The lengths of arrows for environmental variables were multiplied by 1.5 for better visibility. (EC: electrical conductivity (µS cm^−1^), water: soil water content (*m/m*%). Sample identifiers are presented in Table 1). The meaning of the abbreviated substrate names are in the Materials and Methods in Section 2.4.

Statistical analysis of catabolic activity fingerprints was performed using statistical software R v.3.6.0 [28]. Soil basal respiration rates were subtracted from substrate responses to calculate individual substrate induced respiration (SIR) rates. Preliminary plotting revealed a huge difference in respiration rates between sampling sites and weather conditions; therefore, the dataset was standardized by mean sample responses. The effects of vegetation type and weather as grouping factors were tested separately using permutational analysis of variance (PERMANOVA [29]) using the function *adonis* in the vegan package [30], after testing the assumption of multivariate homogeneity of group dispersions, using permutational analysis of multivariate dispersions (PERMDISP [31]) using the function *betadisper* with Bray–Curtis dissimilarities. Soil physical and chemical properties were analyzed in a similar way, with the difference of using Gower distances for PERMDISP. Then, redundancy analysis (RDA) using the *rda* function from the vegan package was applied to determine which soil characteristics had the greatest influence on catabolic activity profiles. The best fitting RDA model was selected based on the Akaike information criterion (AIC) with bidirectional stepwise model selection of the *ordistep* function. Preliminary plotting indicated a nonlinear relationship between EC and soil pH thus EC was log-transformed prior to RDA. The results of the analyses are visualized using the ggplot2 package v.3.3.0 in Figure 2 and Figure 6, and Supplementary Figure S4 [32].

In order to show the similarities among the phylum compositions of the different sodic soil samples, a cluster analysis with the Bray–Curtis similarity index was performed using the PAST software v.3.0 [33]. Similarly to the catabolic profiles, RDA with stepwise model selection was applied to reveal the connection between the distribution of bacterial phyla and environmental variables using the vegan package [30]. In addition, an alternative RDA model including water content was also tested, based on the a priori assumption (confirmed by PCA of the environmental data) that water content was the only soil property differing between the seasons.

## 3. Results

### 3.1. Physical and Chemical Characteristics of the Sodic Soils

The soil moisture content was extremely low in June (the average was 0.16 ± 0.02 *m/m*%), while at least ten-fold higher values (the average was 34.12 ± 2.35 *m/m*%) were measured in September (Table 2). During the extremely dry June sampling, the pH of the sodic grassland soils decreased from highly alkaline to near neutral values in the bare spot (AL)–*Puccinellia* sward (AP)–*Artemisia* alkali steppe (AA)–*Achillea* alkali steppe (AF) sampling site direction (Table 2). During the extreme rainy September sampling, however, the pH values were alkaline at each sampling site and differences between the pH values were much smaller than in June. The electrical conductivity (EC) values showed a decrease both in June and September in the same direction regarding vegetation types as observed for pH. Almost an order of magnitude difference was observed between the maximum EC values at the bare spot (AL) and the minimum EC values at the *Achillea* alkali steppe (AF) in both sampling times. At the same time, soil C_org_ increased in the AL–AP–AA–AF direction. The differences between their minimum and maximum values were higher in June than in September. The CaCO_3_ content of the sodic grassland soils at the bare spot (AL) and *Puccinellia* sward (AP) sampling sites was about one and a half times higher than at the *Artemisia* (AA) and *Achillea* (AF) alkali steppe sampling sites, regardless of the sampling time.

### 3.2. Catabolic Activity Profiles

The mean basal respiration rates of soils measured by MicroResp ranged from 0.11 to 0.33 µg CO_2_-C g^−1^ h^−1^, with the bare spot (AL) samples demonstrating significantly lower values and *Achillea* (AF) alkali steppe samples having higher values than soils from the *Puccinellia* sward (AP) and *Artemisia* (AA) alkali steppe samples. Both basal and SIR rates were greater in June than in September (Figures S1 and S2). Metabolic profiles varied greatly among the four sampling sites. Microorganisms of AL and AP samples utilized lower number of substrates compared to AA and AF samples. 

The results from the PERMANOVA corroborate that both the sampling sites (*p* = 0.001) and sampling dates (*p* = 0.03) had a significant effect on the catabolic profiles (Figure S3, Table S4). Redundancy analysis of the soil chemistry and MicroResp results showed that the primary drivers of catabolic profiles were the EC and soil water content (Figure 2). The overall RDA model was significant (F = 7.68, *p* < 0.01). The constrained variance, which is explained by the final model, was 36.73%, based on the adjusted R^2^ value [34]. The EC contributed mainly to RDA1 (eigenvalue 2.557), as samples separated according to the vegetation types along this axis, although the separation was not complete in the case of the AF and AP samples in June. Differences due to the weather conditions were observed along RDA2, with much less explanatory power (eigenvalue 0.492) than RDA1. The axis RDA2 correlated with the soil water content. The EC and water content were at approximately a right angle, indicating that they were not correlated, and their effects on catabolic profiles were independent.

### 3.3. Diversity of Cultivated Bacterial Strains

**Figure 3:**
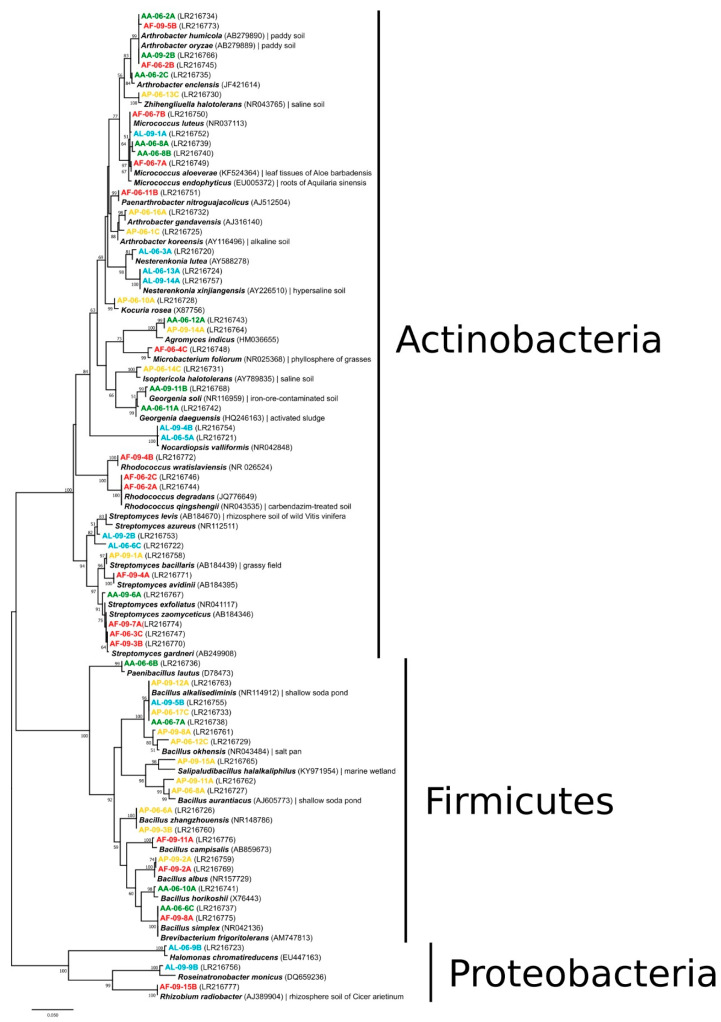
16S rRNA gene sequence-based phylogenetic relations of bacterial strains isolated from the sodic soil samples originated from different alkali vegetation types. GenBank accession numbers are given in parentheses. Only bootstrap values above 50% are shown (500 replications). Bar, 1 nucleotide substitution per 50 nucleotides. For color codes, see Figure 2.

From the cultivated 240 bacterial strains, 58 ARDRA representatives were sequenced and identified (Figure 3). They were closely related to species of the *Agromyces*, *Arthrobacter*, *Brevibacterium*, *Georgenia*, *Isoptericola*, *Kocuria*, *Microbacterium*, *Micrococcus*, *Nesterenkonia*, *Nocardiopsis*, *Paenarthrobacter*, *Rhodococcus*, *Streptomyces* and *Zhihengliuella* (Actinobacteria), *Bacillus*, *Paenibacillus* and *Salipaludibacillus* (Firmicutes), *Halomonas*, *Rhizobium* and *Roseinatronobacter* (Proteobacteria) genera. Members of the phyla Actinobacteria and Firmicutes dominated the strain collection, the while phylum Proteobacteria was represented by single isolates. Altogether 10 species (*Micrococcus luteus*, *Micrococcus yunnanensis*, *Nesterenkonia xinjiangensis*, *Nocardiopsis valliformis*, *Streptomyces gardneri*, *Bacillus alkalisediminis*, *Bacillus aurantiacus*, *Bacillus okhensis*, *Bacillus zhangzhouensis* and *Paenibacillus lautus*) of the 29 and 23 bacterial species identified from the June and September soil samples, respectively, were found at both samplings. The cultivable species diversity was the highest in the genera *Bacillus* and *Streptomyces* with eight and seven different species, respectively. The occurrence of certain species or genera was characteristic of only one sampling site; e.g., *Nesterenkonia xinjiangensis* and *Nocardiopsis valliformis* in the bare spot (AL), *Bacillus alkalisediminis* and *Bacillus aurantiacus* in the *Puccinellia* sward (AP), *Georgenia* for the *Artemisia* (AA) and *Rhodococcus* in the *Achillea* (AF) alkali steppe samples.

### 3.4. Comparison of Sodic Grassland Soil Bacterial Communities by Pyrosequencing

From all four sampling sites and two sampling times, composite sodic grassland soil samples were analyzed by pyrosequencing. The high-throughput DNA analysis of the samples resulted in 29,820 quality-filtered partial 16S rRNA gene sequences with an average length of 320 nucleotides (Table 3). According to Good’s coverage ratios (Table 3), the sequencing depth almost covered the bacterial diversity of the samples. The diversity indices (e.g., inverse Simpson), however, indicated sample-dependent differences. The predicted OTU values (operational taxonomic unit) were on average four times less for the bare spot (AL) and *Puccinellia* sward (AP) samples than for the *Artemisia* (AA) and *Achillea* (AF) alkali steppe samples at both sampling times (Table 3). Similarly, the species richness (e.g., Chao1, ACE) values were, on average, 1.5 times less for the AL and AP than for the AA and AF samples (Table 3).

**Figure 4:**
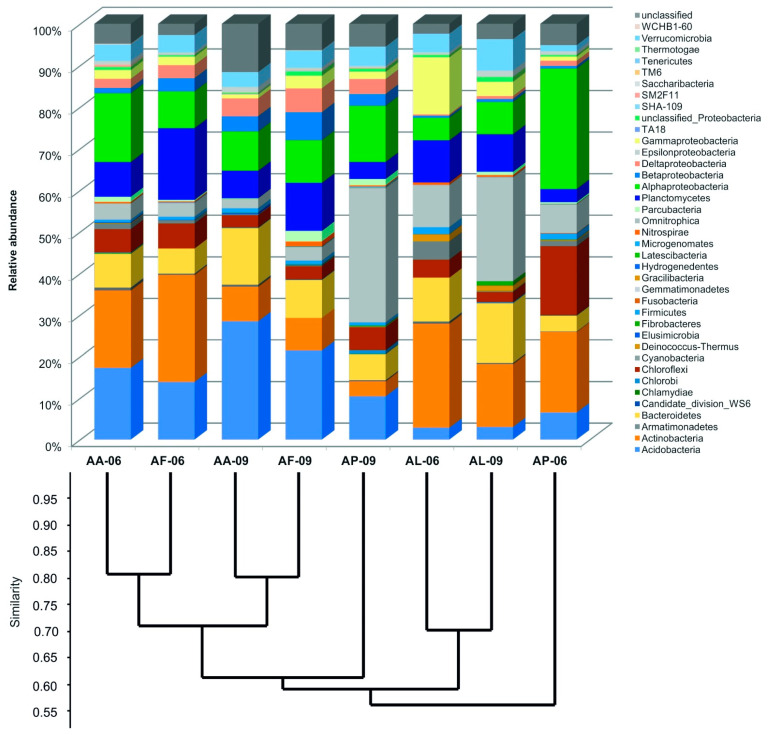
Percentage distribution of 16S rRNA gene amplicon sequences among recognized phyla and candidate divisions together with the results of Bray–Curtis similarity index-based cluster analysis created according to the distribution of OTUs in the sodic soil samples originated from different alkali vegetation. Sample identifiers are presented in Table 1.

Although, no significant differences were found in terms of the numbers of the identified phyla (Table 3), their distribution was different in each sample. Only 10 phyla were shared among the samples from the total of 38 phyla and candidate divisions recovered. Representatives of phyla Proteobacteria, Actinobacteria, Acidobacteria, Gemmatimonadetes and Bacteroidetes were the most abundant (Figure 4). Within the phylum Proteobacteria, phylotypes related to Alphaproteobacteria occupied the highest proportion, except for the June bare spot (AL-06) sample, where Gammaproteobacteria were the most abundant. On average for all samples, the proportions of Acidobacteria (9% vs. 18%), Bacteroidetes (7% vs. 12%), Gemmatimonadetes (6% vs. 14%) and Verrucomicrobia (3% vs. 5%) were lower, while those of Actinobacteria (22% vs. 9%), Proteobacteria (23% vs. 19%) Planctomycetes (9% vs. 7%) and Chloroflexi (9% vs. 3%) were higher in the dry June compared to the wet September samples. 

Based on the results of pyrosequencing data, the average OTU number (defined at 97% similarity) was almost the same in June (540 ± 173) and September (536 ± 113). The number of OTUs, however, was lower in the case of bare spot (AL) and *Puccinellia* sward (AP) samples but higher in the case of *Artemisia* (AA) and *Achillea* (AF) alkali steppe samples in June compared to September (Table 3). Furthermore, the number of OTUs per sample showed an increase in the AL–AP–AA–AF sampling site direction at both sampling times (Table 3). 

**Figure 5:**
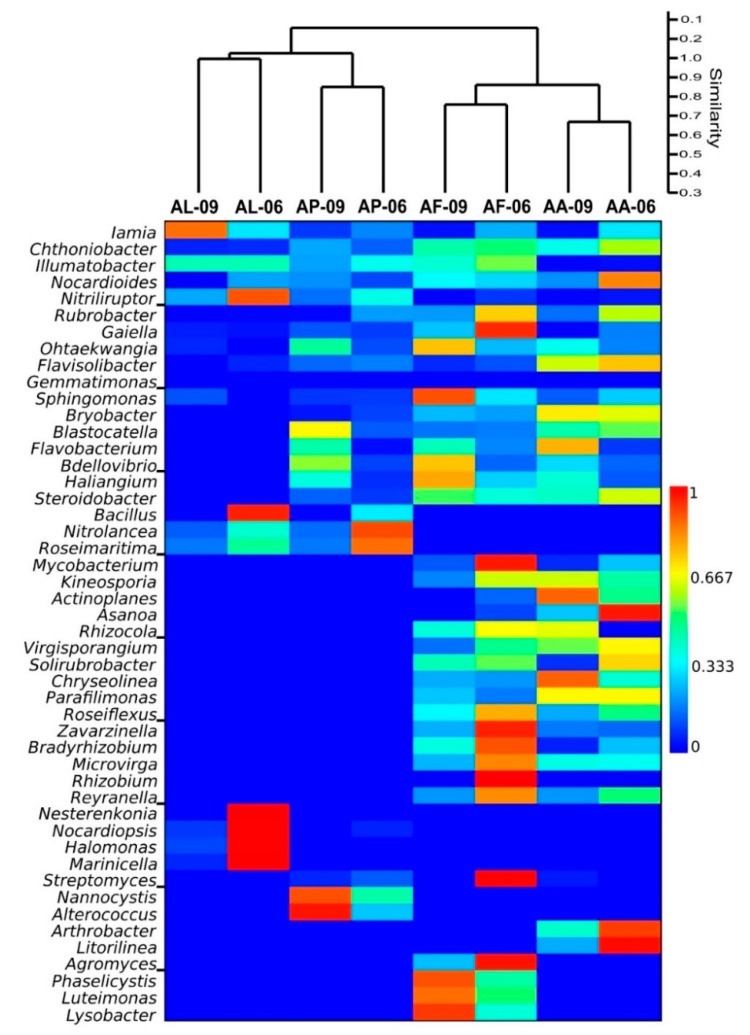
Similarity heatmap of the bacterial genera revealed from pyrosequencing among samples. Sampling sites are given in Table 1.

At genus level, *Iamia* (Actinobacteria) and *Chthoniobacter* (Chthoniobacterales) were identified from all sodic soil samples. Representatives of further genera such as *Illumatobacter* (Acidimicrobiales), *Nocardioides* (Propionibacteriales), *Nitriliruptor* (Nitriliruptorales), *Rubrobacter* (Rubrobacterales) and *Gaiella* (Gaiellales) within the phylum Actinobacteria, *Ohtaekwangia* (Cytophagales) and *Flavisolibacter* (Chitinophagales) within the phylum Bacteroidetes, *Gemmatimonas* (Gemmatimonadales, Gemmatimonadetes), *Defluviicoccus* (Rhodospirillales) and *Sphingomonas* (Sphingomonadales) within the phylum Proteobacteria were detected in all but one sample (Figure 5). 

Sequences closely related to the genera *Bryobacter* (Bryobacterales) and *Blastocatella* (Blastocatellales) within the phylum Acidobacteria, *Flavobacterium* (Flavobacteriales) within the phylum Bacteroidetes, and *Bdellovibrio* (Bdellovibrionales), *Haliangium* (Myxococcales) and *Steroidobacter* (Steroidobacter_o) within the phylum Proteobacteria were retrieved from all alkali vegetation types except from the bare spot (AL). Sequences belonging to genera *Bacillus* (Bacillales) within the phylum Firmicutes, *Nitrolancea* (Sphaerobacterales) within the phylum Chloroflexi and *Roseimaritima* (Planctomycetales) within the phylum Planctomycetes were characteristic of bare spot (AL) and *Puccinellia* sward (AP) samples. Representatives of genera *Candidatus* Solibacter (Solibacterales) within the phylum Acidobacteria, *Mycobacterium* (Corynebacteriales), *Kineosporia* (Kineosporiales), *Actinoplanes*, *Asanoa*, *Rhizocola*, *Virgisporangium* (Micromonosporales) and *Solirubrobacter* (Solirubrobacterales) within the phylum Actinobacteria, *Chryseolinea* (Cytophagales) and *Parafilimonas* (Chitinophagales) within the phylum Bacteroidetes, *Roseiflexus* (Chloroflexales) within the phylum Chloroflexi, *Zavarzinella* (Planctomycetales) within the phylum Planctomycetes, and *Bradyrhizobium*, *Microvirga, Rhizobium* (Rhizobiales) and *Reyranella* (Rhodospirillales) within the phylum Proteobacteria were present only in *Artemisia* (AA) and *Achillea* (AF) alkali steppe samples (Figure 5).

The following genera were characteristic of one vegetation type: *Nesterenkonia* (Micrococcales) and *Nocardiopsis* (Streptosporangiales) within the phylum Actinobacteria, *Halomonas* (Oceanospirillales) and *Marinicella* (Marinicella_o) within the phylum Proteobacteria for bare spot (AL), *Streptomyces* (Streptomycetales) within the phylum Actinobacteria, *Nannocystis* (Myxococcales) within the phylum Proteobacteria and *Alterococcus* (Opitutales) within the phylum Verrucomicrobia for *Puccinellia* sward (AP), *Arthrobacter* (Micrococcales) and *Candidatus* Microthrix (Acidimicrobiales) within the phylum Actinobacteria and *Litorilinea* (Caldilineales) within the phylum Chloroflexi for *Artemisia* (AA) alkali steppe, and *Agromyces* (Micrococcales) within the phylum Actinobacteria, and *Phaselicystis* (Myxococcales), *Luteimonas* and *Lysobacter* (Lysobacterales) within the phylum Proteobacteria for *Achillea* (AF) alkali steppe samples (Figure 5).

**Figure 6:**
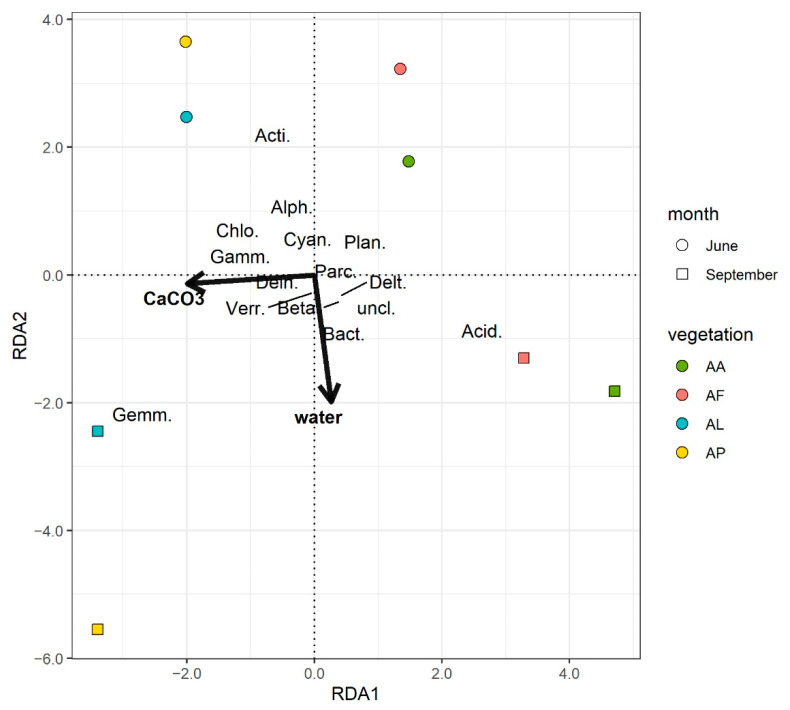
Redundancy analysis plot of the final model for the phylogenetic (NGS) data. All taxa were included in the analysis, but for better readability only those that had at least a 0.1 absolute value for both axes are depicted on the plot. The lengths of the arrows for the environmental variables were also doubled for visibility purposes. CaCO_3_: percentage of CaCO_3_, water: water content of the soil samples (*m/m*%), Acid.: Acidobacteria, Acti.:Actinobacteria, Bact.: Bacteriodetes, Chlo.: Chloroflexi, Cyan.: Cyanobacteria, Dein.: Deinococcue-Thermus, Gemm.: Gemmatoidetes, Parc.: Parcubacteria, Plan.: Planctomycetes, Alph.: Alphaproteobacteria, Beta.: Betaproteobacteria, Delt.: Deltaproteobacteria, Gamm.: Gammaproteobacteria, Verr.: Verrucomicrobia, uncl.: Unclassified bacteria.

The only environmental variable that proved to significantly influence the community structure was soil CaCO_3_, as found with the RDA by stepwise selection. Although the overall model was significant (F = 2.67, *p* < 0.05), the explanatory power of the model was found to be weak applying the correction suggested by Peres-Neto et al. [34] with an adjusted R^2^ value of 0.192. Plotting the first two residual axes (Figure S4) showed a clear distinction of the samples according to the wet and dry samplings which was not explained by this model. In an alternative RDA model, the soil water content was included in addition to the soil CaCO_3_. It was based on a priori knowledge regarding the environmental data, as only water content differed significantly between samplings. This model was found to be more significant (F = 2.90, *p* < 0.01), and it better explained the differences in the community structures (R^2^_adj_ = 0.3519). Therefore, this model was selected as the final RDA model (Figure 6). Similarly to the results of catabolic profiles, samples were separated along RDA1 (eigenvalue = 133.17) based on vegetation type, although the difference between AA and AF as well as between AL and AP samples was low. The samples correlated to the CaCO_3_ content along the RDA1 axis. Differences according to weather extremes (the soil water content) were observed along the RDA2 axis (eigenvalue = 98.96). The soil CaCO_3_ and water contents were at approximately a right angle, indicating their independent effects on the bacterial community structures.

## 4. Discussion

Worldwide soil salinization and its consequences are among the most important issues not only from an ecological and environmental but also from an agricultural point of view [35,36,37,38,39,40,41]. It is known that plants including species of halophytic vegetation can alter the taxonomic and metabolic diversity of microorganisms in their environments by changing the rhizosphere soil chemical composition compared to the bulk soils. The so-called plant growth promoting rhizobacteria (PGPR) living in close association with the plant roots can facilitate the salt tolerance of their hosts in many ways, e.g., by producing ACC deaminase enzyme, secreting phytohormones, controlling the Na^+^ homeostasis, etc. [40,42]. Therefore, the question arises as to how the compositional complexity and the potential metabolic activity of sodic soil microbial communities can change owing to the naturally varying salt stress occurring in the saline soils of different natural halophytic plant associations.

Micromosaic-like vegetation types often develop in the saline areas of the KNP (Hungary) due to the varying soil salinity, sodicity and alkalinity. The effects of dry–wetting cycles on plant associations were examined in detail [12,13], and the largest differences were found in the soil electrical conductivity values and moisture contents due to the primary salinization process taking place as a consequence of natural dry–wetting cycles and groundwater movements. The present study confirmed the above-mentioned previous results and demonstrated that the increased but varying soil salinity and alkalinity through the alkali vegetation types has considerable effects on the activity and composition of the sodic soil bacterial communities.

The results of the MicroResp data indicated a close relationship between the alkali vegetation types and community level physiological profiles (CLPPs) of the studied microbial communities (Figure 2). Differences in the CLPPs were pronounced in September and barely noticeable in June. Distinctions among the sampling sites could be explained mainly by the different EC of the soil samples. This is in accordance with the results of a previous examination of the mid-term vegetation changes in the Apaj area, where the type of plant coverage showed the best correlation with the salinity (EC) [43]. 

All the studied carbon sources induced the lowest catabolic activities in the bare spot (AL) samples, which had the highest pH and EC values. Higher catabolic activities were observed both in June and September in the lower salinity soil samples. Comparing the substrate-induced respiration to the basal respiration of microbial communities, vegetation type- and soil chemistry-dependent changes were also observed previously [44]. The low metabolic activity of microbial communities in highly saline soils could originate from the small and stressed microbial biomass [45]. In the present study, the high soil pH could also be a potential stressor.

It is interesting to note that higher catabolic activity was detected in the dry (June) sodic soils compared to the wet (September) soils (Figures S1–S3). Studying the resilience of soil microbial communities using the MicroResp method, Bérard et al. [46] observed that soils had higher basal respiration rates after drought and heat stress. The differences were explained by two mechanisms of the so-called “Birch-effect”; one of them was a “substrate supply” due to the release of soil organic material in the drying rewetting cycle, the other was the reuse possibility of organic materials originated from dead and lysed microbes because of the “environmental stress” [46]. They also described that both desiccation and heat stress influenced the catabolic profiles. Therefore, it is possible that not only the drying rewetting cycle, but also the extreme heat stress during our early summer sampling changed the metabolic activities of microbial communities. 

Irrespective of the medium composition used for cultivation, our strain collection contained two main types of bacterial species: (1) members of typical plant-associated bacteria (e.g., *Micrococcus aloeverae*, *Micrococcus endophyticus*, *Microbacterium foliorum*, *Streptomyces levis*, *Streptomyces bacillaris*, *Rhizobium radiobacter*), and (2) representatives of halotolerant, halophile and/or alkaliphile species (e.g., *Zhihengliuella halotolerans*, *Arthrobacter koreensis*, *Nesterenkonia xinjiangensis*, *Isoptericola halotolerans*, *Bacillus aurantiacus*, *Bacillus alkalisediminis*) described from saline, hypersaline and/or alkaline habitats. In a previous study, the rhizosphere soil of different halophytes (*Bolboschoenus maritimus* and *Puccinellia limosa*) near the soda ponds of the KNP was also found to be dominated by isolates of haloalkaliphilic *Bacillus*, *Nesterenkonia* and *Halomonas* species [22]. The widely distributed *Bacillus* species can frequently be isolated from saline and alkaline environments (including sodic soils); two alkaliphilic and moderately halophilic species (*B. aurantiacus* and *B. alkalisediminis*)—representatives of which occurred in all sampling sites and times except one in the present study—were described from the soda ponds of the KNP, Hungary [47,48]. *Bacillus* species have versatile metabolism and significant biotechnological ability as they can participate in the phosphate solubilization, carbonate precipitation and nitrogen-fixation, synthesis of various extracellular enzymes, antimicrobial compounds and phytohormones [10,41,49,50,51]. *Streptomyces* species are also typical soil inhabitants which can tolerate extreme environmental factors (e.g., salinity of soils) and are able to produce several extracellular enzymes and bioactive molecules (e.g., phytohormones, siderophores, antibiotics) whereby they can also be considered as PGPR [51,52,53]. Therefore, the high taxonomic diversity of cultivated *Bacillus* and *Streptomyces* species in the studied sodic soils can not only be related to their capability to tolerate harsh environmental conditions but also the fact that the high salt concentrations can increase the synthesis of their secondary metabolites [54]. The representatives of the genera *Nesterenkonia* and *Halomonas* were also among the members previously isolated from the sodic soils in the KNP, Hungary [22], but they were also detected from the saline-alkaline pastures located in Daqing, China [55]. In the present study, isolates belonging to different aerobic saprotrophic *Arthrobacter* species were isolated from each sodic soil samples except for the lowest humus-containing bare spot. Similarly, *Arthrobacter* isolates were found to be one of the main cellulose-degraders in the alkaline-saline chinampa soils with high humus content [51]. In addition, other aerobic chemoorganotrophic isolates also have broad metabolic potential, and some strains of the identified species can decompose specific organic compounds (e.g., degradation of polychlorinated biphenyls and low-molecular polycyclic aromatic hydrocarbons by *Rhodococcus degradans*, carbendazim by *Rhodococcus qingshengii* and monochlorophenols by *Georgenia daeguensis*) based on literature data [56,57,58]. 

The high-throughput amplicon sequencing (NGS) resulted in a distinction of bacterial community structures according to the CaCO_3_ content of the sodic soil samples characteristic of the alkali vegetation types (Figure 5). The soils of the *Artemisia* and *Achillea* alkali steppe vegetation (AA and AF) had similar community compositions, while the *Puccinellia* sward (AP) had similar bacterial communities to the bare spot (AL) soils (Figure 5). These differences correlated mainly with the CaCO_3_ content of the soils. Weather extremes also contributed the separation of the soil bacterial communities between AA and AF samples and between AL and AP samples correlated mainly with the soil water content.

The decrease in the alkalinity and salinity of soils from June to September could be responsible for the increase in the calculated species richness and diversity index values, as well as the detected OTU numbers (Table 3) in the case of bare spot and *Puccinellia* sward soils (AL and AP). The species richness and diversity index changed to a greater extent for the higher salinity and alkalinity soils (AL and AP) than the *Artemisia* and *Achillea* alkali steppe vegetation (AA and AF) during the studied drying–rewetting period. The weather extremes experienced during the samplings seemed to have little effect on both the phylum- and order-level compositions of the bacterial communities, but rather on the relative abundance of the community members. Similar results, such as relatively drying–rewetting-independent bacterial community compositions, were revealed from grass soils of a Mediterranean-type ecosystem (Santa Ynez, CA, USA) [59].

The distribution of the most abundant bacterial phyla (Proteobacteria, Actinobacteria, Acidobacteria, Gemmatimonadetes and Bacteroidetes) revealed from the KNP sodic soil of different alkali vegetation types were partly overlapping with those found in the mosaic vegetation-covered saline soil of Piana del Signore, a semiarid natural Mediterranean area located in Sicily, Italy [36]. The phyla Proteobacteria, Actinobacteria, Acidobacteria and Bacteroidetes were also found to be predominant in a meta-analysis study on publicly available bacterial sequences originated from saline soils [10]. Studying the responses of soil microorganisms to extreme desiccation and rewetting in Californian semiarid grasslands, the phyla Acidobacteria and Actinobacteria were found to be the most abundant and potentially the most active members of the bacterial communities [60]. The desiccation and rewetting driven changes in the relative abundances of phyla Acidobacteria and Actinobacteria were similar in the present study as well. Among the bacterial phyla explored, the highest rate but opposite direction changes were observed in these two phyla. The proportion of Acidobacteria increased while Actinobacteria decreased in the wet (September) samples compared to the dry (June) samples (Figure 4). The relative abundance of the phylum Acidobacteria in our sodic soil samples, however, showed difference not only according to weather extremes but also by vegetation types. The percentage distribution of the phylum Acidobacteria was much lower, while that of the phylum Gemmatimonadetes was much higher in the high salinity bare spot and *Puccinellia* sward samples than in the *Artemisia* and *Achillea* alkali steppe samples. In a comparative high-throughput sequencing study performed on saline alkaline soils with different land use, a similar strong correlation was observed among soil properties (e.g., pH, water content, electrical conductivity and organic content) and land use as well as the relative abundance of bacterial phyla, as the proportion of Actinobacteria and Gemmatimonadetes was higher in the most alkaline and saline grassland soil than in the agricultural and forest soils [55]. 

In accordance with the results of cultivation, the phylotypes revealed by NGS were closely related to plant-associated bacterial genera or halophilic and/or alkaliphilic genera previously described from alkaline-saline habitats (e.g., soils, seawater, soda lakes). These bacteria can decompose different organic materials and can participate in the nitrogen and phosphorous cycles of the soils, as well. From the *Puccinellia* sward (AP) samples, high abundance of sequences was assigned to the genus *Defluviicoccus* (Rhodospirillales)*,* a glycogen-accumulating alphaproteobacterium commonly found in the activated sludge of wastewater treatments plants [61]. Sequences affiliated with the genus *Sphingomonas* (Sphingomonadales) were present in each sodic soil sample. Members of this genus are widely distributed in nature, including different plant root systems [62] and contaminated soils [63], where they can take part in the decomposition of various organic compounds (e.g., polycyclic aromatic hydrocarbons). The genera of *Bradyrhizobium, Microvirga* revealed by NGS and *Rhizobium* explored by cultivation (Rhizobiales) as potential nitrogen-fixers were detected from the sodic soils of *Artemisia* and *Achillea* alkali steppe vegetation (AA and AF samples). Based on the 16S rRNA gene sequence similarities, sequences assigned to the genus *Gemmatimonas* (Gemmatimonadales) are among the widely distributed members of different soil inhabitants, including extreme environments [10,64,65], as was found in the present study. The type species of the genus *Gemmatimonas* was described as a polyphosphate-accumulating bacterium from an anaerobic–aerobic sequential batch reactor used enhanced biological phosphorus removal conditions for wastewater treatment [66]. In this study, sequences affiliated with the genus *Lysobacter* (Lysobacterales) were detected only in the *Achillea* alkali steppe vegetation; however, it was found among the dominant populations in the Jerusalem artichoke saline rhizosphere soil [67]. Species of the genus *Lysobacter* can be characterized by special metabolic activities (e.g., antibiotic and extracellular enzyme production); therefore, they can be potentially used in controlling plant diseases [68]. Several genera (e.g., *Nocardioides, Nitriliruptor, Rubrobacter, Gaiella*) were ubiquitous in the sodic soil of the KNP alkali vegetation types, while others (e.g., *Mycobacterium, Kineosporia, Actinoplanes, Asanoa, Rhizocola, Virgisporangium, Solirubrobacter*) were present mainly in the *Artemisia* and *Achillea* alkali steppe vegetation (AA and AF). Previously, representatives of the genera *Solirubrobacter, Sphingomonas, Nocardioides, Arthrobacter,* etc. were also found to be the most abundant in agricultural and forest land uses, while *Nitriliruptor* in the saline-alkaline land in Daqing (China) using pyrosequencing of bacterial 16S rRNA genes [55].

## 5. Conclusions

The differences in the alkali vegetation types due to the soil chemistry (mainly the CaCO_3_ and EC values) had a greater impact on the metabolic activity and taxonomic composition of microbial communities than the weather extremes, despite the small geographical distances of the studied sodic soils. The effects of extreme aridity and moisture were reflected in the changes in the relative abundance of certain taxonomic groups (primarily Acidobacteria and Actinobacteria). The highly different bacterial communities of soda soils with different alkali vegetation types were dominated by alkaliphilic and/or halophilic and plant-associated bacterial taxa revealed by both cultivation and pyrosequencing.

## Figures and Tables

**Table 1:** Sampling sites, geographical coordinates and vegetation characteristics.

	Bare Spot	*Puccinellia* Sward	*Artemisia* Alkali Steppe	*Achillea* Alkali Steppe
Sample Identifier	AL-06; AL-09	AP-06; AP-09	AA-06; AA-09	AF-06; AF09
Geographic coordinates	47°05′11.6″ N, 19°05′54.4″ E	47°05′08.5″ N, 19°06′07.1″ E	47°05′09.4″ N, 19°06′02.6″ E	47°05′11.4″ N, 19°05′53.1″ E
Natural plant association	Lepidio crassifolii—Champhorosmetum annuae	Lepidio crassifolii—Puccinellietum limosae	Artemisio santonici—Festucetum pseudovinae	Achilleo setaceae—Festucetum pseudovinae
Plant coverage	16%	68%	42%	90%
Dominant plant species	*Lepidium crassifolium*, *Champhorosma annua*	*Puccinellia limosa*	*Artemisia maritima*, *Plantago maritima*, *Festuca pseudovina*	*Festuca pseudovina*, *Achillea setacea*
Other plant species (accompanying/sporadic)	*Puccinellia limosa*	*Phragmites australis*, *Lepidium crassifolium*, *Plantago maritima*	*Podospermum canum*, *Puccinellia limosa*, *Agropyron repens*	*Cerastium pumilum*, *Plantago lanceolate*, *Koeleria cristata*, *Agropyron repens*, *Lotus corniculatus*, *Thymus pannonicus*, *Potentilla argentea*, *Trifolium repens*

**Table 2:** Physical and chemical parameters of the sodic soil samples from different alkali vegetation types in June (06) and in September (09). Data represent the mean values ± S.D (*n* = 3).

	Bare Spot		*Puccinellia* Sward		*Artemisia* Alkali Steppe		*Achillea* Alkali Steppe	
Sample Identifier	AL-06	AL-09	AP-06	AP-09	AA-06	AA-09	AF-06	AF-09
pH_H2O_	10.3 ± 0.1	10.3 ± 0.1	8.9 ± 0.6	9.5 ± 0.2	8.3 ± 0.3	9.9 ± 0.1	7.4 ± 0.2	8.0 ± 0.2
EC	3800 ± 834	2147 ± 114	1196 ± 220	1131 ± 46.5	562 ± 202	592 ± 28.7	325 ± 35.7	284 ± 22.9
Salt	0.55 ± 0.23	0.25 ± 0.02	0.14 ± 0.03	0.13 ± 0.01	0.05 ± 0.04	0.13 ± 0.02	<0.02	<0.02
C_org_	0.4 ± 0.1	0.3 ± 0.0	1.3 ± 0.3	0.6 ± 0.1	1.7 ± 0.3	1.1 ± 0.2	2.6 ± 0.2	1.6 ± 0.5
CaCO_3_	22.3 ± 4.1	20.9 ± 3.0	23.2 ± 0.7	22.5 ± 1.0	12.5 ± 4.2	12.5 ± 0.9	15.9 ± 2.9	15.6 ± 2.2

EC (µS cm^−1^); Salt, C_org_ and CaCO_3_ in %.

**Table 3:** Pyrosequencing read numbers, coverage, OTU numbers, richness estimators, diversity index and number of different taxa for bacterial communities from the sodic soil samples originated from different alkali vegetation types. Sample identifiers are presented in Table 1.

		Sample Identifier							
		AL-06	AL-09	AP-06	AP-09	AA-06	AA-09	AF-06	AF-09
Total high-quality sequences		4493	3571	4580	2658	3144	5958	3350	2066
Good’s coverage		99.89%	99.69%	99.80%	99.74%	99.87%	99.97%	99.70%	99.18%
Number of OTUs		366	382	417	520	669	624	707	618
Species richness	Chao1	429.1	447.3	497.2	543.1	721.0	855.0	775.1	618.3
	ACE	431.0	451.2	500.8	545.6	722.3	855.7	778.5	624.5
Diversity index	Inverse Simpson	55.9	86.9	17.2	69.1	242.7	173.6	235.3	265.7
Number of identified	phyla	17	20	25	22	23	23	19	24
	orders	93	86	101	90	99	100	102	97
	genera	141	126	174	161	204	216	219	194

## Data Availability

The 16S rRNA gene sequences were submitted to the GenBank under the accession numbers LR216720-LR216777 for isolated bacterial strains. Raw genomic sequence reads are available in the NCBI Sequence Read Archive as BioProject accession PRJNA316799. Soil chemical data and microresp data presented in this study are available in the supplementary file S1.

## Supplementary Materials

The following are available online at https://www.mdpi.com/article/10.3390/microorganisms9081673/s1, Figure S1: Mean basal respiration rates (±S.D., *n* = 3; µg CO_2_-C·g soil^−1^·h^−1^) of the soil samples from the four sites, Figure S2: Means (±S.D., *n* = 3) of the summarized substrate induced respiration rates (µg CO_2_-C·g soil^−1^·h^−1^) of the soil samples after addition of 15 different substrates from the four sites in two samplings, June and September, Figure S3: Mean substrate induced respiration (SIR) rates (±S.D., *n* = 3; µg CO_2_-C·g soil^−1^·h^−1^) of the soil samples after addition of the various carbon sources, Figure S4: Plot of the first two axis of residual variance (PCA) from the RDA model of the bacterial phylogenetic (NGS) and environmental data (CaCO_3_) with *ggplot2 package* v.3.3.0, Table S1: ANOVA results of the soil chemical properties, Table S2: ANOVA results of the soil basal respiration (Figure S1), Table S3: ANOVA results of the cumulative substrate induced respirations (Figure S2), Table S4: PERMANOVA results based on Bray–Curtis dissimilarities using abundance data for MicroResp (Figure S3), File S1: Data S1: Soil chemical data, Data S2: microResp data.
