# Supplementary material for: Variation in Sodic Soil Bacterial Communities Associated with Different Alkali Vegetation Types
Source: Microorganisms. 2021 Aug 6;9(8):1673. doi: 10.3390/microorganisms9081673 (PMC8402138; doi:10.3390/microorganisms9081673)
Supplement: Supplementary file 1 [file microorganisms-09-01673-s001.zip › microorganisms-1285503-supp/sodicsoil_MI_ESM_v7_szkt.pdf]

Microorganisms

Electronic Supplementary Material for

Variation in Sodic Soil Bacterial Communities Associated with Different Alkali Vegetation Types

Andrea K. Borsodi[1*], Márton Mucsi[1,2], Gergely Krett[1], Attila Szabó[3], Tamás Felföldi[1], Tibor Szili-Kovács[2,*]

1 Department of Microbiology, ELTE Eötvös Loránd University, Pázmány P. sétány 1/C,H-1117 Budapest, Hungary; borsodi.andrea@ttk.elte.hu; tamas.felfoldi@ttk.elte.hu; gergely.krett@ttk.elte.hu
2 Institute for Soil Sciences, Centre for Agricultural Research, Herman Ottó út 15, H-1022 Budapest, Hungary; mucsi.marton@atk.hu; szili-kovacs.tibor@atk.hu
3 Institute of Aquatic Ecology, Centre for Ecological Research, Karolina út 29, H-1113 Budapest, Hungary; szabo.attila@ecolres.hu
* Correspondence: borsodi.andrea@ttk.elte.hu (AK.B.); Tel.: (+3613812177); szili-kovacs.tibor@atk.hu (T. Sz-K.); Tel: (+36309617452)

Table S1. ANOVA results of the soil chemical properties considering the month (June and September) and vegetation type 1) bare spot (AL), 2) Puccinellia sward (AP), 3) Artemisia alkali steppe (AA), 4) Achillea alkali steppe (AF).

**Soil pH$_{H2O}$**					
	Df	Sum Sq	Mean Sq	F value	Pr(>F)
Month	1	3.315	3.315	36.333	1.76e-05 ***
Vegetation	3	21.216	7.072	77.504	9.57e-10 ***
MonthxVeg.	3	2.103	0.701	7.682	0.00212 **
Residuals	16	1.460	0.091		

**Soil EC**					
	Df	Sum Sq	Mean Sq	F value	Pr(>F)
Month	1	738855	738855	5.704	0.029599 *
Vegetation	3	22713826	7571275	58.451	7.67e-09 ***
MonthxVeg.	3	4559198	1519733	11.733	0.000258 ***
Residuals	16	2072496	129531		

**Soil Corg**					
	Df	Sum Sq	Mean Sq	F value	Pr(>F)
Month	1	2.190	2.1901	35.530	2.00e-05***
Vegetation	3	9.104	3.0348	49.233	2.66e-08***
MonthxVeg.	3	1.185	0.3948	6.406	0.00468**
Residuals	16	0.986	0.0616		

**CaCO3**					
	Df	SumSq	Mean Sq	F value	Pr(>F)
Month	1	8.4	8.39	1.084	0.313
Vegetation	3	409.2	136.41	17.624	2.52e-05***
MonthxVeg.	3	30.0	9.99	1.291	0.312
Residuals	16	123.8	7.74		

**Soil moisture**					
	Df	Sum Sq	Mean Sq	F value	Pr(>F)
Month	1	752.0	752.0	160.513	9.33e-10 ***
Vegetation	3	244.0	81.3	17.362	2.76e-05 ***
MonthxVeg.	3	56.2	18.7	4.001	0.0266 *
Residuals	16	75.0	4.7		

Df: degrees of freedom, Sum Sqs: sum of squares, Mean Sqs: mean of squares, Significance codes: *** p<0.001 ** p<0.01, * p<0.05. EC = Electrical conductivity.

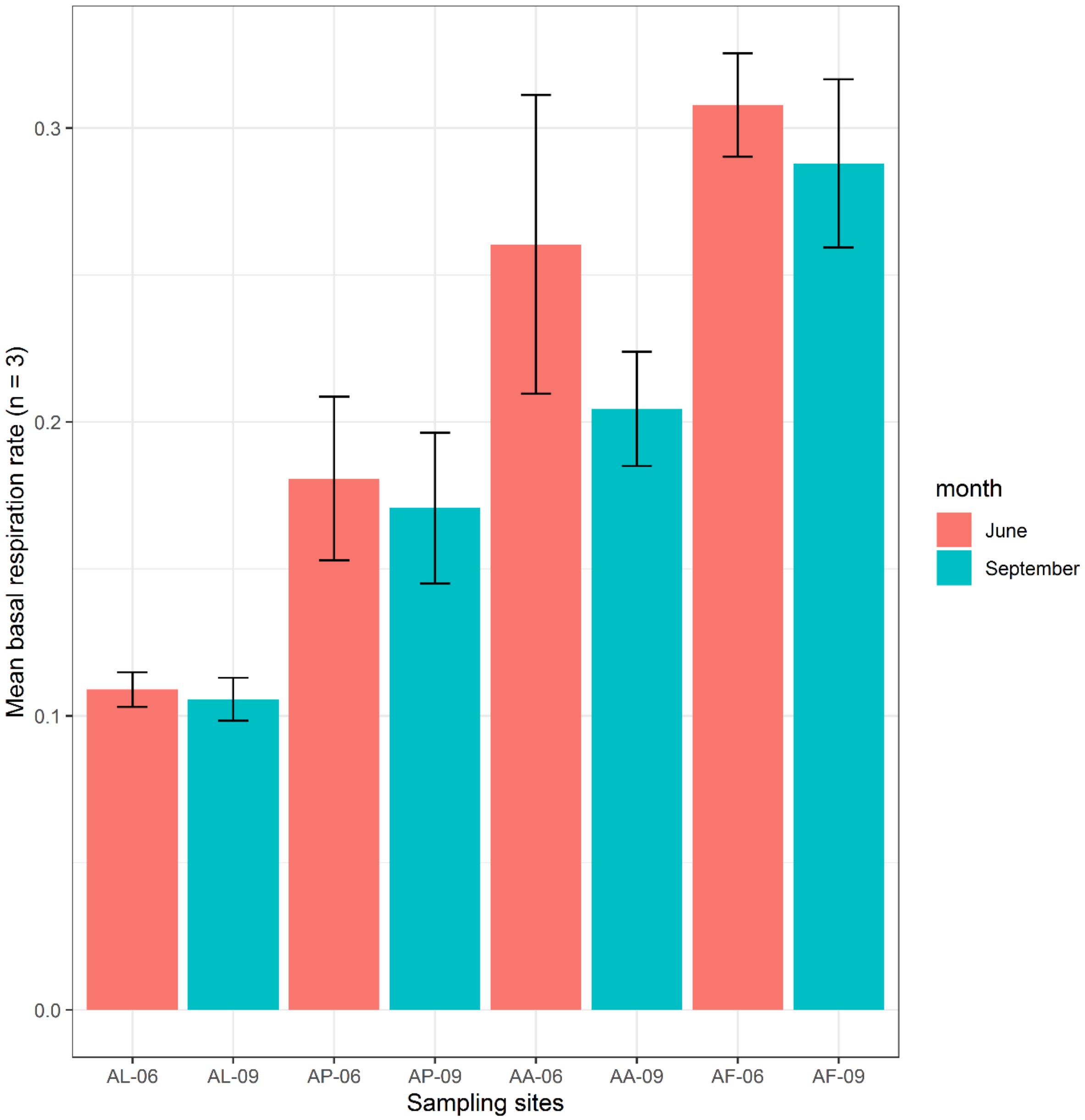

Figure S1. Mean basal respiration rates (± S.D., n = 3; µg CO_2-C*g soil^{-1}*h^{-1}) of the soil samples from the four sites. Samples are bare spot (AL), Puccinellia sward (AP), Artemisia alkali steppe (AA) and Achillea alkali steppe (AF), with June (-06) and September (-09) sampling are presented. Basal soil respiration was assessed from MicroResp short term incubation (5-hours) without substrate addition, only distilled water was added in the same volume as the other wells with substrate solutions. 6 parallel wells per sample was used.

Table S2. ANOVA results of the soil basal respiration (Figure S1) considering the month (June and September) and vegetation type 1) bare spot (AL), 2) Puccinellia sward (AP), 3) Artemisia alkali steppe (AA), 4) Achillea alkali steppe (AF).

	Df	Sum Sq	Mean Sq	F value	Pr(>F)
Month	1	0.00298	0.00298	4.249	0.0559 .
Vegetation	3	0.11862	0.03954	56.406	9.95e-09 ***
MonthxVeg.	3	0.00247	0.00082	1.176	0.3501
Residuals	16	0.01122	0.00070		

Df: degrees of freedom, Sum Sqs: sum of squares, Mean Sqs: mean of squares, Significance codes: *** p<0.001 ** p<0.01, * p<0.05, . p<0.1.

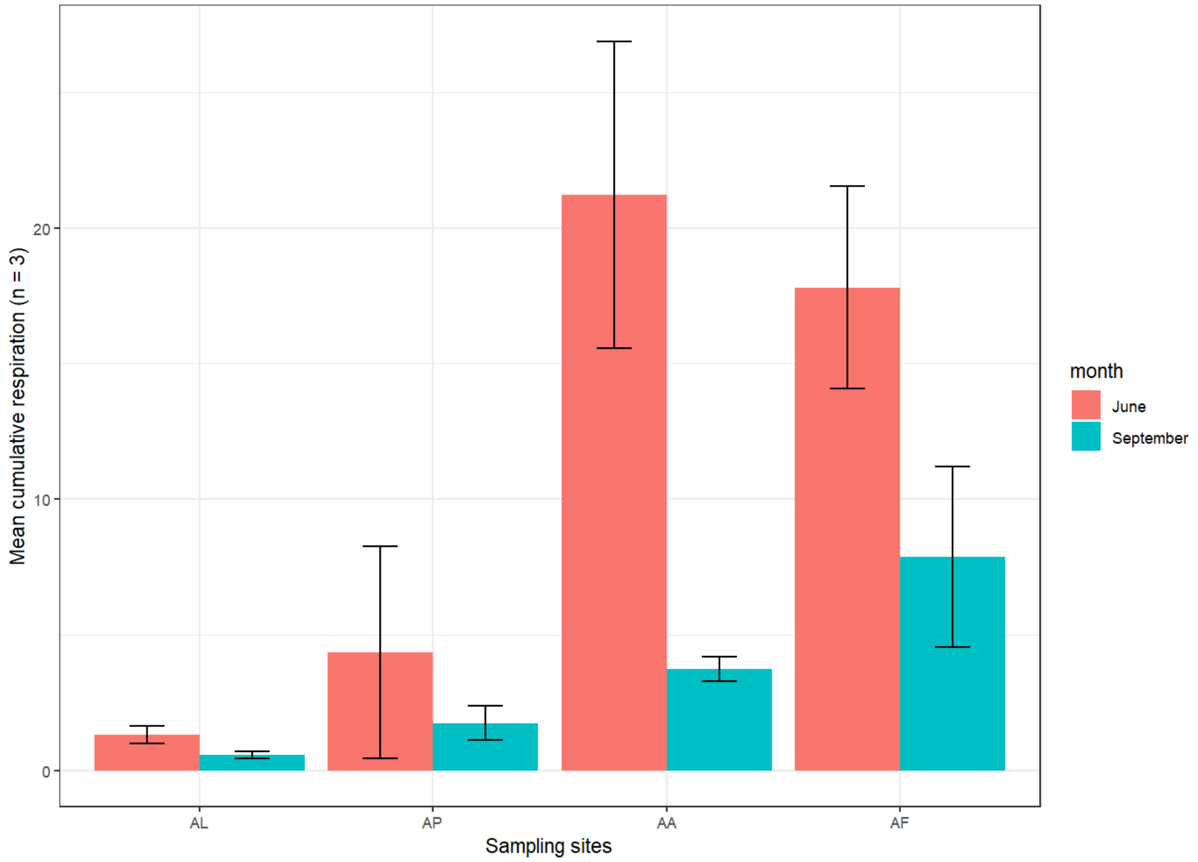

Figure S2. Means (± S.D., n = 3) of the summarized substrate induced respiration rates (μg CO_2-C*g soil^{-1}*h^{-1}) of the soil samples after addition of 15 different substrates from the four sites in two samplings, June and September. Samples are bare spot (AL), Puccinellia sward (AP), Artemisia alkali steppe (AA) and Achillea alkali steppe (AF) in June and September. Substrate induced respiration was assessed by MicroResp short term incubation (5-hours) with 15 different substrate addition.

Table S3. ANOVA results of the cumulative substrate induced respirations (Figure S2) considering the month (June and September) and vegetation type 1) bare spot (AL), 2) Puccinellia sward (AP), 3) Artemisia alkali steppe (AA), 4) Achillea alkali steppe (AF).

	Df	Sum Sq	Mean Sq	F value	Pr(>F)
Month	1	387.0	387.0	46.38	4.19e-06 ***
Vegetation	3	975.3	325.1	38.97	1.39e-07 ***
MonthxVeg.	3	286.7	95.6	11.45	0.000293 ***
Residuals	16	133.5	8.3		

Df: degrees of freedom, Sum Sqs: sum of squares, Mean Sqs: mean of squares, Significance codes: *** p<0.001 ** p<0.01, * p<0.05, . p<0.1.

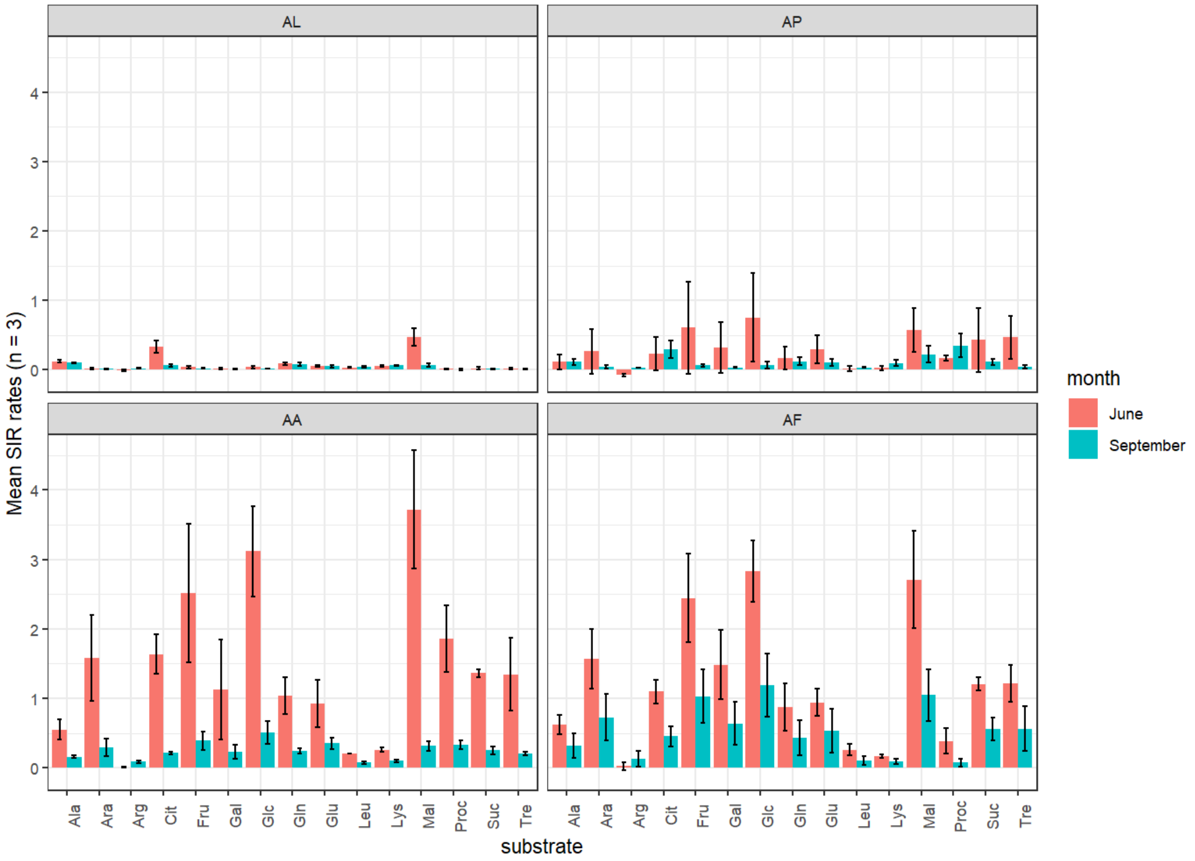

Figure S3. Mean substrate induced respiration (SIR) rates (± S.D., n = 3; µg CO_2-C*g soil^{-1}*h^{-1}) of the soil samples after addition of the various carbon sources. Samples are bare spot (AL), Puccinellia sward (AP), Artemisia alkali steppe (AA) and Achillea alkali steppe (AF) in June and September. Substrate induced respiration was assessed by MicroResp short term incubation (5-hours) with 15 different substrate addition (25 µL/well, 6 replicate wells in each plate). Added substrates were D-galactose (Gal), trehalose (Tre), L-arabinose (Ara), D-glucose (Glc) and D-fructose (Fru) in 80 mg mL^{-1}, citric acid (Cit), DL-malic acid (Mal), Na-succinate (Suc), L-alanine (Ala) and L-lysine (Lys) in 40 mg mL^{-1}, L-glutamine (Gln) in 20 mg mL^{-1}, and L-leucine (Leu), L-arginine (Arg) protocatechuic acid (Proc) and L-glutamic acid (Glu) in 12 mg mL^{-1}.

Table S4. PERMANOVA results based on Bray-Curtis dissimilarities using abundance data for MicroResp (Figure S3) for a) month (June and September), b) vegetation types: 1) bare spot (AL), 2) Puccinellia sward (AP), 3) Artemisia alkali steppe (AA), 4) Achillea alkali steppe (AF).

	Df	SumSqs	MeanSqs	Pseudo-F	R2	P
a)						
month	1	0.224120055	0.22412	3.471991	0.136306	0.036*
Residuals	22	1.420119313	0.064551		0.863694	
Total	23	1.644239368			1	
b)						
vegetation	3	0.950914199	0.316971	9.143513	0.578331	0.001*
Residuals	20	0.693325169	0.034666		0.421669	
Total	23	1.644239368			1	

Df: degrees of freedom, SumSqs: sum of squares, MeanSqs: mean of squares, Pseudo-F: F value by permutation, asterisks indicate statistical significance with P<0.05, P-values are based on 999 permutations.

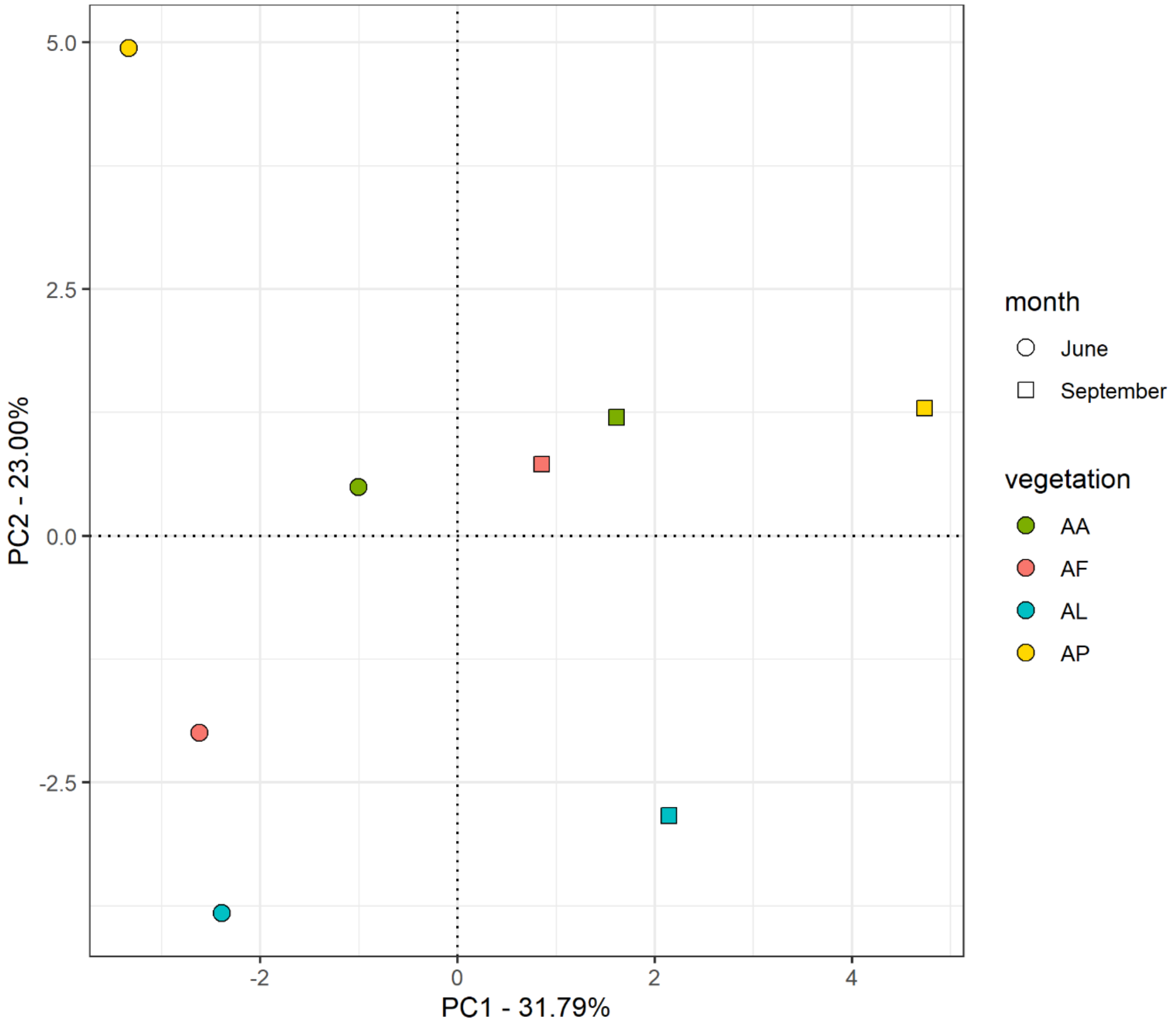

Figure S4. Plot of the first two axis of residual variance (PCA) from the RDA model of the bacterial phylogenetic (NGS) and environmental data ($CaCO_3$) with *ggplot2 package* v.3.3.0 (Wickham, 2016). Samples are bare spot (AL), Puccinellia sward (AP), Artemisia alkali steppe (AA) and Achillea alkali steppe (AF) in June and September sampling.

Reference

Wickham, H. *ggplot2: Elegant Graphics for Data Analysis.* Springer-Verlag, New York, USA, 2016. https://ggplot2.tidyverse.org.
